# Understanding tolerance to cell wall–active antibiotics

**DOI:** 10.1111/nyas.14541

**Published:** 2020-12-03

**Authors:** Tobias Dörr

**Affiliations:** ^1^ Weill Institute for Cell and Molecular Biology, Department of Microbiology, and Cornell Institute of Host–Pathogen Interactions and Disease Cornell University Ithaca New York

**Keywords:** antibiotic tolerance, phenotypic tolerance, beta lactams, autolysins, peptidoglycan

## Abstract

Antibiotic tolerance—the ability of bacteria to survive for an extended time in the presence of bactericidal antibiotics—is an understudied contributor to antibiotic treatment failure. Herein, I review the manifestations, mechanisms, and clinical relevance of tolerance to cell wall–active (CWA) antibiotics, one of the most important groups of antibiotics at the forefront of clinical use. I discuss definitions of tolerance and assays for tolerance detection, comprehensively discuss the mechanism of action of β‐lactams and other CWA antibiotics, and then provide an overview of how cells mitigate the potentially lethal effects of CWA antibiotic–induced cell damage to become tolerant. Lastly, I discuss evidence for a role of CWA antibiotic tolerance in clinical antibiotic treatment failure.

## Graphical abstract

Herein, I review the manifestations, mechanisms, and clinical relevance of tolerance to cell wall–active (CWA) antibiotics, one of the most important groups of antibiotics at the forefront of clinical use. I discuss definitions of tolerance and assays for tolerance detection, comprehensively discuss the mechanism of action of β‐lactams and other CWA antibiotics, and then provide an overview of how cells mitigate the potentially lethal effects of CWA antibiotic–induced cell damage to become tolerant. Lastly, I discuss evidence for a role for CWA antibiotic tolerance in clinical antibiotic treatment failure.

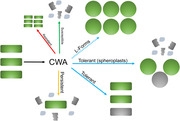

## Part I. Introduction

The discovery, development, and clinical implementation of antibiotics stands as one of the greatest achievements of the 20th century. They have enabled the treatment of myriad infectious diseases and facilitated important life‐saving advances in medicine, such as organ transplantation. Among the most important are cell wall–active (CWA) antibiotics, which selectively inhibit the synthesis or turnover of this essential bacterial structure. These agents include, *inter alia*, the β‐lactams (e.g., penicillins, cephalosporins, carbapenems, and monobactams), glycopeptides (e.g., vancomycin, teicoplanin, telavancin, oritavancin, and dalbavancin), and fosfomycin. The history describing their discovery and clinical development is the source of legend. These accounts range from the serendipitous, yet Argus‐eyed, discovery of penicillin by A. Fleming in the 1920s who noted the inhibitory activity on *Staphylococcus aureus* on an agar plate contaminated with the mold *Penicillium notatum*,^1^ to the exotic with the discovery by E. Kornfeld in the 1950s of compound 05865, that is, vancomycin (named from the word “vanquish”), produced by an isolate, *Streptomyces orientalis* (now *Amycolatopsis orientalis*), recovered from a soil sample from Borneo.^2^

However, these and related compounds have often been deployed with abandon and little consideration to the ease with which resistance can emerge. The emergence and increasing occurrence of antibiotic resistant organisms is a serious public health concern and places a substantial burden on the healthcare system and society in general. The Centers for Disease Control and Prevention estimates that more than 2 million antibiotic resistant infections occur in the United States each year, resulting in at least 23,000 deaths, $20 billion in excess direct healthcare costs, and $35 billion in lost productivity.^3^ Clearly, frank antibiotic resistance is a global threat; however, antibiotic tolerance (defined below) is believed to contribute to both treatment failure and the development of overt antibiotic resistance. Despite seminal work in the 1970s and 1980s that focused on CWA antibiotic tolerance, this remains underappreciated and understudied (e.g., a PubMed search for “antibiotic tolerance” yields 1/60th of the number of articles unearthed with the search term “antibiotic resistance”). Therefore, the primary motivating factor for this review is to illuminate the topic of tolerance of CWA antibiotics and to inspire the scientific community to address this important and fascinating subject.

### Definitions and measurements of tolerance

**Figure 1:**
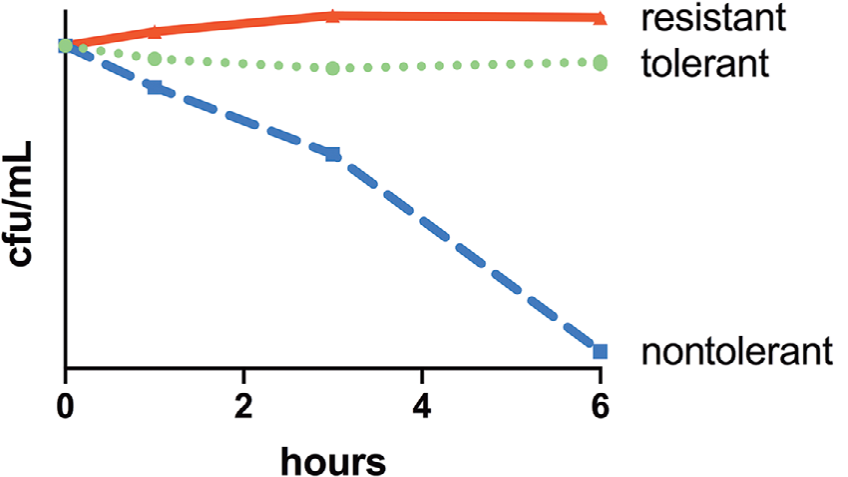
Time‐kill experiment illustrating the distinction between CWA antibiotic resistance, tolerance, and lack of tolerance at high inoculum. Shown are real data from an actual experiment where either a resistant isolate of *Klebsiella pneumoniae* (carrying the carbapenemase KPC‐1), a tolerant isolate of the same species (*K. pneumoniae* 1084), or a nontolerant *E. coli* isolate were exposed to the carbapenem antibiotic meropenem. The resistant isolate divides and thus increases in live cell counts, while the tolerant isolate neither grows nor significantly dies and the nontolerant isolate declines in viability by several orders of magnitude. Note that what is shown here represents exposure to antibiotics at high inoculum, where a very high number of tolerant cells remains viable.

Antibiotic tolerance remains a poorly defined term. Typically, it is used to express that bacteria remain viable (but unable to grow) for extended periods after exposure to an antibiotic typically considered bactericidal (Fig. 1). This is in clear contrast to resistance, which is defined as the ability to proliferate in the presence of an antibiotic (quantifiable by well‐defined measures, such as the zone of inhibition around an antibiotic‐containing disk, or the minimum inhibitory concentration, MIC). I herein treat tolerance as largely separate from persistence, which is defined as a small fraction of a bacterial population that is completely refractory to killing by bactericidal antibiotics. Numerous excellent reviews are available concerning persistence,^4^, ^5^, ^6^ which will not be discussed specifically in this review. However, as there is an overlap between persistence (small subpopulation of bacterial cells that survive antibiotic exposure) and tolerance (whole population of bacterial cells slowly dying), inclusion of persisters to some degree is necessary. Indeed, persistence has previously been defined as *heterotolerance*, that is, a subcategory of tolerance.^4^

Numerous attempts have been made to quantify tolerance in a clinically meaningful way. The earliest studies utilized some measure of the absolute survival numbers of a population after antibiotic exposure for a certain timeframe, with arbitrary cutoff points for what was considered tolerant; for example, >100 colony forming units (cfu)/mL after 24‐h incubation with antibiotic^7^ or 2% survival after 24 hours.^8^ A more widely established measure for tolerance is the minimum bactericidal concentration (MBC), typically reported in its relationship with the MIC. Tolerance breakpoints were often arbitrarily set at MBC/MIC >10–100 (Ref. 9). For MBC determinations, bacterial cultures are exposed to increasing concentrations of an antibiotic, followed by the determination of viability after some fixed time of incubation (typically 24 hours).^10^, ^11^ Usually, killing at first increases with antibiotic concentration, followed by a plateau where little additional killing is observed. In some instances, higher antibiotic concentrations effect less killing (termed the *Eagle effect* or *paradoxical effect*, which can be observed with certain β‐lactam antibiotics^12^). The MBC is then (again, somewhat arbitrarily) defined as the minimal concentration of antibiotic that kills 99.9% of bacteria. Several problems with the MBC metric for assessing tolerance were reported early on. Unlike MIC determinations, MBCs were found to be highly variable between different growth media and culture volumes used in these assays,^9^, ^13^, ^14^, ^15^, ^16^ adversely affecting its usefulness for clinical studies. Further, some bactericidal antibiotics never yield an MBC against some bacterial species (even if they are fully susceptible, but less than 99.9% of the population is killed).^17^, ^18^ Most importantly, when considering the phenomenology of tolerance, a bacterial isolate with a low MBC might still be considered tolerant if it takes an extended time to reach 99.9% of killing. For example, an isolate that experiences a 1000‐fold drop in viability within 1 h at a certain antibiotic concentration would arguably be less tolerant than an isolate that takes 24 h to reach the same level of killing, yet both might have the same MBC. As a potential way to circumvent this problem, another proposed tolerance measure uses the time needed to kill 99–99.9% of bacteria; that is, the minimum duration of killing (MDK_99_) as a readout for tolerance.^19^, ^20^ Lastly, Goessens *et al*. proposed the use of the tolerance percentage, which is measured by conducting an MBC experiment, but then reporting the percent survival of the bacterial population at the beginning of the plateau, that is, the survival fraction at the antibiotic concentration above which no further killing is observed.^8^ As such, the tolerance percentage would likely be a useful quantifier for persistence but not necessarily for tolerance since the latter is generally defined through a temporal aspect (“slower” killing). Therefore, the MDK_99_ is probably the most relevant measurement to capture the full breadth of tolerance phenotypes.

What almost all tolerance tests have in common is that unlike the gold standard MIC, they are too cumbersome and time‐consuming to be routinely conducted in a clinical laboratory. This problem has been recognized and two tests have thus been established to measure tolerance by the appearance of colonies in the zone of growth inhibition around an antibiotic‐containing disk upon prolonged incubation. This is accomplished either by adding, post‐incubation, an enzyme that degrades the antibiotic,^21^ or, in its more recent iteration from Nathalie Balaban's group, by adding nutrients and relying on antibiotic dissipation through diffusion to allow for regrowth of rare survivors (TDtest^22^). While these tests are only semiquantitative, for example, provide readouts of “no colonies” versus “few colonies” versus “many colonies,” they could potentially be compatible with a clinical microbiology laboratory workflow, opening up new avenues to include tolerance testing in the clinical practice.

## Part II. Mechanisms of tolerance

### Mechanism(s) of action of CWA antibiotics

**Figure 2:**
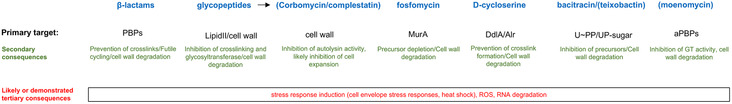
Summary of the main classes of CWA antibiotics and their mechanism of action. Antibiotics not used clinically are in parentheses. ROS, reactive oxygen species; GT, glycosyltransferase.

To understand mechanisms of tolerance, one must first understand the mechanism of action of CWA antibiotics (summarized in Fig. 2); for that, an excursion into bacterial cell wall synthesis and turnover is necessary. Most bacteria surround themselves with a cell wall, which is either a thin layer (∼2–6 nm) covalently linked to an outer membrane (Gram‐negative bacteria) or a thick layer (∼50–60 nm) covalently linked with other polysaccharides (e.g., teichoic acids (TAs)) that often have regulatory functions.^23^, ^24^ In both Gram‐negative and ‐positive bacteria, the cell wall mainly comprises peptidoglycan (PG), a polymer of [*N*‐acetyl‐glucosamine]‐[*N*‐acetylmuramic acid] (NAG–NAM) residues that have short oligopeptide chains attached to the NAM residues. The NAG‐NAM disaccharide unit is almost invariant across bacterial species (with the exception of condition‐specific modifications like *O*‐acetylation^25^, ^26^), while the oligopeptide chain is highly variable depending on species and growth conditions.^24^ However, some commonalities, grounded in biochemical necessity, exist. For example, in all bacteria, the peptide sidechain contains d‐amino acids; the third position is occupied by a d‐amino acid that possesses free amine groups (typically diaminopimelic acid (DAP) or lysine (Lys)) and positions 4 and 5 consist of two d‐alanine (d‐Ala) residues in the vast majority of bacterial species.^24^, ^27^

The basic building block for the cell wall is called lipid II and represents an NAG–NAM disaccharide with a pentapeptide sidechain and a C_55_ lipid anchor (undecaprenol phosphate (UP)). This precursor is produced in the cytoplasm by an assembly line of so‐called Mur enzymes, which first produce an NAM monosaccharide that is sequentially outfitted with amino acids to build the oligopeptide chain. The resulting NAM‐peptide is then attached to UP‐pyrophosphate (UPP) and subsequently joined with an NAG residue to form lipid II. Lipid II is translocated into the periplasm by dedicated flippase enzymes (MurJ and Amj^28^, ^29^, ^30^), where it is then used to assemble the final cell wall in two major reactions. The pyrophosphate bond in lipid II is used to provide energy for a glycosyltransferase reaction (**GT reaction**); that is, the polymerization of the disaccharides into polysaccharide strands of varying lengths.^31^ This reaction liberates the UPP carrier, which is first dephosphorylated by several phosphatases^32^, ^33^, ^34^, ^35^ and then recycled for another round of lipid II production in the cytoplasm.^36^, ^37^ The second reaction is transpeptidation (**TP reaction)**, which exploits the energy of the d‐Ala–d‐Ala link to effect covalent linkage between a free amine group of DAP or Lys in the third position of the oligopeptide sidestem and the subterminal d‐Ala residue of an adjacent PG strand.^31^ Variations of this theme exist, with often elaborate peptide crossbridges between the Lys‐d‐Ala bond in Gram‐positive bacteria, as well as direct DAP–DAP crosslinks in both Gram‐negatives and ‐positives.^24^, ^27^

**GT reactions** can be catalyzed by three different types of synthases: the class A penicillin‐binding proteins (aPBPs), the shape, elongation, division, and sporulation (SEDS) proteins RodA and FtsW, as well as monofunctional transglycosylases with unclear physiological significance.^38^, ^39^, ^40^, ^41^, ^42^ **TP reactions** are catalyzed by aPBPs and monofunctional class B PBPs (bPBPs); the latter typically associate with SEDS proteins to catalyze cell wall assembly.^39^, ^40^, ^41^ A special type of TP reaction (e.g., catalyzing crosslinking between DAP–DAP) is mediated by l,d transpeptidases.^43^, ^44^, ^45^ Why bacteria encode separate biochemical entities catalyzing essentially the same reactions is poorly understood, but aPBPs appear to have a general (nonspecialized) PG synthesis and perhaps repair function,^46^, ^47^, ^48^ while bPBP–SEDS pairs have more specialized functions in cell elongation and division.^39^, ^40^, ^41^ A balance between aPBPs and bPBP‐SEDS activities is also important for cell width and cell wall turnover homeostasis in *Bacillus subtilis*.^49^, ^50^

While the PG cell wall is essential for maintenance of structural integrity and thus a relatively rigid structure, it is also remarkably dynamic. A large number of often biochemically redundant enzymes termed *autolysins* are able to cut and process PG. For a detailed discussion of these enzymes, several excellent reviews are available (e.g., see Refs. 51, 52, 53, 54). The physiological functions of these cutting processes include creating gaps for insertion of new PG during cell elongation,^55^, ^56^, ^57^ facilitating daughter cell separation after cell division,^58^, ^59^, ^60^, ^61^, ^62^, ^63^, ^64^ insertion of macromolecular transenvelope complexes into the cell wall,^65^, ^66^ and PG recycling.^67^ Autolysins are organized into four major families. The amidases cleave between the oligopeptide sidechain and the polysaccharide backbone. The endopeptidases (EPs) cut the intermolecular oligopeptide crosslinks between DAP_3_ and d‐Ala_4_ (d,d EPs), between DAP_3_–DAP_3_, as well as intramolecular DAP_3_‐d‐Glu_2_ bonds (d,l EPs), or within the peptide bridge elaborated by some Gram‐positive bacteria.^68^ The lytic transglycosylases (LTGs) cut within the polysaccharide backbone in a nonhydrolytic, intramolecular nucleophilic attack mechanism that generates characteristic saccharide‐anhydro residues,^69^ while the similar muramidases cut the backbone through a hydrolytic mechanism. Lastly, the carboxypeptidases (which cleave off terminal amino acids from the oligopeptide sidestems) are often grouped with the autolysins, though their cleavage activity does not directly result in cell wall integrity failure.^54^, ^70^

The amidases are active during cell division and have a function in daughter cell separation, which is understood in great mechanistic detail.^61^, ^63^ EPs have a demonstrated, yet less well‐understood role in cell elongation and division.^55^, ^56^, ^57^, ^71^, ^72^ The LTGs and muramidases facilitate insertion of transenvelope complexes, PG recycling, daughter cell separation, and toxin secretion.^64^, ^65^, ^67^, ^73^, ^74^ The carboxypeptidases have a poorly understood role in cell shape generation and in the regulation of PG synthesis.^70^, ^75^, ^76^, ^77^, ^78^ Since autolysin‐mediated cleavage processes are potential threats to cellular structural integrity, they are likely carefully controlled or balanced by efficient cell wall synthesis at all times, and this has indeed been demonstrated for amidases and to some degree for EPs.^62^, ^63^, ^79^, ^80^, ^81^, ^82^ Understanding the role and cleavage specificities of autolysins will be important to understand the mechanism of action of CWA antibiotics in the next sections.

#### Mechanism of action of β‐lactam antibiotics

The mechanism of action of β‐lactam antibiotics is simple: they inhibit the TP active site of PBPs. Following the resulting interruption of PG assembly, the cell wall is usually degraded, causing either lysis and death, or the emergence of viable, cell wall–deficient forms.^17^, ^83^, ^84^, ^85^, ^86^, ^87^, ^88^ However, considering the downstream consequences of β‐lactam–PBP interactions in more detail gives rise to considerable complexity. First, not all β‐lactams have the same affinity for all PBPs. In extreme cases, specific PBPs are exclusively inhibited by specific antibiotics (e.g., bPBP2 in many Gram‐negative bacteria, which is inhibited by mecillinam, or bPBP3, which in many bacteria is inhibited by aztreonam or cephalexin), but most β‐lactams bind to multiple PBPs with different affinities.^89^, ^90^, ^91^, ^92^, ^93^ Even at concentrations above the MIC, the consequences of β‐lactam exposure can thus be a function of the compound's concentration, rather than causing a simple dichotomy of growth versus death. For example, imipenem inhibits bPBP2 primarily at concentrations just above the MIC, but all PBPs at higher concentrations,^94^ favoring dramatically different cell fates (see below). Affinities can vary between bacterial species too. The Gram‐negative bPBP2 inhibitor mecillinam mentioned above specifically inhibits the class A PBPs in *Streptococcus pneumoniae*,^92^ and cefsulodin inhibits both aPBPs in *Escherichia coli*, but only aPBP1B in *Vibrio cholerae*.^95^ The physiological importance of specific β‐lactam–PBP affinities becomes apparent when considering the second complication of β‐lactam mechanism of action: inhibition of different PBPs can have drastically different consequences for cells. Inhibition of the cell division–specific bPBP3 by aztreonam results in filamentation and slow death, and inhibition of PBP2 results in continued cell wall synthesis, but an inability to divide and maintain rod shape and thus delayed lysis and death. By contrast, inhibition of the aPBPs generally results in catastrophic structural integrity failure and rapid cell lysis and death.^90^, ^94^, ^96^ In the model organism *E. coli*, where most work on β‐lactam susceptibility has been conducted, the most rapid lysis is observed when aPBPs plus one or more bPBPs are inhibited simultaneously.^94^

The last complication of β‐lactams is the historic observation that β‐lactam exposure can result in two separate death pathways, a lytic pathway and a nonlytic pathway. This was first observed by Alexander Tomasz's group in the 1970s where an autolysin‐deficient mutant of *S. pneumoniae* did not lyse in the presence of penicillin, but still died.^97^ Similarly, isolates of various *Streptococcus* species differ in their lytic responses: *Streptococcus sanguis* neither lyses nor dies (but is growth‐inhibited), *Streptococcus pyogenes* does not lyse, but dies, and *S. pneumoniae* both lyses and dies in the presence of penicillin.^98^ Thus, lysis and death induced by β‐lactam antibiotics are separable phenomena. The question of what kills lysis‐deficient, β‐lactam–exposed cells was reilluminated by Thomas Bernhardt's group in 2014. Here, the paradoxical observation that the bPBP2 inhibitor mecillinam still killed cells with genetic backgrounds where bPBP2 is nonessential, led to the realization that β‐lactam exposure does not merely result in PG synthesis inhibition and activation of autolysins. In addition to inducing cell wall degradation, inhibition of the TP active site promotes the continuous generation of long, uncrosslinked PG strands, which are immediately degraded by LTGs in a process termed *futile cycling*.^99^ This runaway process in turn likely results in precursor depletion (especially depletion of the limited UP membrane carrier, which is shared with other essential biochemical pathways) as well as unnecessary energy expenditure, and significantly contributes to cell death.^99^ Consistent with such a model, overexpression of aPBP mutants with inactivated TP domains induces lysis in *E. coli*,^100^ putatively also due to futile cycling.

#### Glycopeptide antibiotics

The glycopeptide antibiotics are high molecular weight antibiotics that are generally only active against Gram‐positive bacteria as due to their large size they do not significantly penetrate the outer memberane of most Gram‐negative bacteria.^101^ What all glycopeptides have in common is their ability to bind tightly to PG or PG precursors, though the specific binding region and thus killing potency can vary. Vancomycin and related glycopeptides (e.g., oritavancin) bind tightly to the d‐Ala‐d‐Ala end of stem oligopeptides and thus primarily inhibit the TP reaction through steric obstruction of PG synthase–substrate binding.^102^ However, vancomycin binding can also inhibit GT activity due to its affinity to the pentapeptide in lipid II.^103^, ^104^ Ramoplanin has a high affinity for the sugar portion of lipid II, essentially sequestering this essential building block from PG synthases and thereby inhibiting PG assembly (primarily by preventing GT activity).^105^ The newly discovered glycopeptides complestatin and corbomycin exhibit general PG binding properties (likely binding the polysaccharide backbone), which obstructs the activity of autolysins.^106^ Lastly, some newer generation glycopeptides (e.g., telavancin) may have additional mechanisms of action like membrane disruption and depolarization.^107^

#### Precursor synthesis and recycling inhibitors

Several clinically used antibiotics inhibit PG precursor synthesis and recycling. Fosfomycin, which is used to treat infections due to members of the Enterobacterales,^108^ covalently modifies and inhibits MurA, which catalyzes the first step of PG precursor synthesis.^109^ Inhibition ultimately results in precursor depletion, precluding cell wall assembly. d‐cycloserine is a structural d‐Ala analog that inhibits alanine racemase as well as d‐Ala‐d‐Ala ligase,^110^ resulting in the production of a lipid II species that lacks the d‐Ala‐d‐Ala cap and can thus not be used for canonical d,d crosslinking by the PBPs. Bacitracin and teixobactin are cyclic peptides with a unique mechanism of action: both bind to the UPP carrier molecule. Bacitracin sequesters the free form of UPP,^111^ thus inhibiting the dephosphorylation and recycling of this essential precursor. Ultimately, cell wall assembly fails due to the cell's inability to translocate precursors across the cytoplasmic membrane. Teixobactin likely interacts with UPP when linked with a saccharide residue (as found in lipid II and lipid III)^112^ and this compound inhibits PG synthesis as well as other UP‐dependent synthesis pathways,^113^ potentially through sequestration of building blocks in supramolecular complexes.^114^

#### Primary consequences of CWA antibiotic exposure

Why PG degradation and lysis is a consequence of exposure to CWA antibiotics is poorly understood in detail for most bacteria; however, autolysins are clearly the main effectors of this process,^88^, ^115^ and this was recognized as early as 1957.^116^ In *S. pneumoniae*, the amidase LytA is the sole contributor to penicillin‐induced lysis. Under normal growth conditions, LytA is sequestered by lipoteichoic acids (LTAs).^23^, ^117^ Exposure to penicillin results in the degradation of an LTA synthase, the lack of which then favors the production of cell wall–associated TAs (WTAs) rather than LTAs. WTA recruits LytA, resulting in cell wall degradation, lysis, and death.^118^ Consequently, a ∆*lytA* mutant fails to lyse in the presence of penicillin.^118^, ^119^, ^120^ Similarly, mutants in the major autolysin of *S. aureus*, the bifunctional muramidase/amidase Atl, exhibit reduced death and lysis in the presence of CWA antibiotics,^121^, ^122^ suggesting some commonality with the *S. pneumoniae* system. The identification of a single autolysin that is required for CWA antibiotic–induced lysis (like in *S. pneumoniae*) is unusual. Most bacteria contain a multitude of autolysins with their specific contributions to the lysis process unknown. Species‐specific differences may also exist. In *E. coli*, products of LTG and EP‐mediated PG degradation are observed after β‐lactam exposure,^123^, ^124^ and the amidases appear to be major contributors to lysis.^61^ In *V. cholerae*, EPs mediate initial cell wall degradation after exposure to penicillin, while the sole amidase is not required for the degradation process itself, but determines the location of its initiation (with a shift from septal blebbing to lateral wall blebbing in a ∆*amiB* mutant).^17^ Interestingly, exposure to the aPBP glycosyltransferase inhibitor moenomycin results in the degradation of the PG sacculus but does not yield LTG products in *E. coli*,^124^ suggesting that LTG activity is not actually required for sacculus destruction under all conditions. Likely, the appearance of LTG breakdown products thus depends on active GT activity (see “futile cycling” discussed above) by the Rod system and/or aPBPs.

#### Other downstream events of PG synthesis inhibition

All things considered, exposure to CWA antibiotics is a traumatic event for any bacterial cell, as these antibiotics eventually cause the destruction of the cell wall by autolysins. Thus, not surprisingly, exposure to such agents results in various sequelae that reflect distinct cellular responses as well as purely physical consequences of massive cell wall damage. Exposure to β‐lactams has been tied to the generation of reactive oxygen species (ROS),^125^ or not;^126^, ^127^ activation of iron uptake;^128^ changes in TCA cycle intermediate concentrations;^129^ induction of the heat shock response;^130^, ^131^ induction of the SOS DNA damage response;^132^, ^133^ RNA degradation;^116^, ^134^ and stringent response (SR) induction.^135^ In principle, any or all of these processes may contribute to some degree to β‐lactam–mediated cell death (or tolerance, as shall be considered later), but their detailed contributions have remained poorly understood.

### Environmental factors promoting tolerance

A well‐recognized hallmark of antibiotic efficacy is its strong dependence on environmental factors,^136^, ^137^ and this is especially true for CWA antibiotic tolerance. For example, pH,^138^, ^139^ the presence of certain d‐amino acids in the growth medium^140^ or coculture with other bacteria,^141^ can drastically affect CWA antibiotic tolerance through poorly understood mechanisms. Tolerance can be a function of media composition as well: planktonic cells of *Mycobacterium abscessus*, for example, exhibit high tolerance to the β‐lactam antibiotic cefoxitin (and other antibiotics) in standard laboratory growth media, but not in artificial cystic fibrosis sputum medium.^142^

Environmental variables that affect growth are particularly relevant for CWA antibiotic tolerance. Since cell death and lysis induced by CWA antibiotics relies on active PG turnover as detailed above, a reduction or cessation of cell wall modifications should result in increased tolerance. One way of achieving this is to stop growing, and especially to stop cell division, which appears to be a particularly dynamic (and thus vulnerable) aspect of cell wall synthesis and turnover. Processes associated with cell division are, indeed, a major contributor to β‐lactam–induced lysis, at least in *E. coli*.^143^, ^144^, ^145^ Further, it is well established that the degree and rate of death induced by β‐lactam antibiotics strongly correlates with growth rate;^146^, ^147^ that is, slowly growing cells are killed less rapidly than fast growing cells. Not surprisingly then, tolerance is often enhanced by environmental conditions that favor slow growth. As such, entry into stationary phase, nutrient/metal depletion, and growth in biofilms are often associated with high tolerance to β‐lactams.^142^, ^148^, ^149^, ^150^, ^151^ In addition, reduced growth might be the underlying cause of the inoculum effect, that is, the observation that usage of high inocula for killing assays results in drastically increased MBCs.^152^ Lastly, slow growth may also underlie the observed tolerance of respiration‐defective small colony variants in multiple bacterial species.^153^

Slow growth is likely a major contributor to tolerance during infection, given the inhospitable environment infecting bacteria often find themselves in. Nutrient depletion, cell damage by immune functions, and transition metal sequestration affect growth rate in the host and not surprisingly, many infections indeed yield nongrowing or slowly growing cells.^154^, ^155^ This was demonstrated in model “tissue cage” infections, where a porous plastic body is inoculated with a bacterial suspension and inserted subcutaneously into experimental animals to emulate infections associated with medical devices.^156^ In a rat infection model of *S. aureus*, tissue cages were mostly populated by nongrowing cells (defined by their failure to resume growth within 2 h of subculture into Mueller Hinton Broth (MHB)) that were 1000‐ to 10,000‐fold more tolerant to oxacillin and vancomycin than bacteria grown solely in MHB.^157^ *Salmonella typhimurium* growing in macrophages becomes highly tolerant against a variety of antibiotics (including cell wall–active compounds),^158^ and this has also been proposed to be a consequence of slow/nongrowth under these nonoptimal conditions.^149^, ^151^, ^158^ Similarly, tolerance can also be caused simply by coadministration of antibiotics that stop or slow down growth.^159^ For example, antagonism between chloramphenicol (CHL) and penicillin is a well‐known *in vivo* and *in vitro* phenomenon^20^, ^115^, ^160^ and likely relies on the protective slowdown/shutdown of growth induced by inhibitors of translation. Likewise, erythromycin can treacherously induce vancomycin tolerance in *S. pneumoniae*, confounding the interpretation of tolerance assays.^161^, ^162^, ^163^

However, it is important to note that slow/nongrowth is actually neither necessary nor sufficient for tolerance to PG‐acting compounds. Slowly or nongrowing *Borrelia burgdorferi* cells (the etiological agent of Lyme disease) are effectively killed by vancomycin and ceftriaxone in stationary phase;^164^ conversely, *V. cholerae* fails to be appreciably killed even when exposed to β‐lactams during optimal growth in exponential phase.^17^, ^165^, ^166^ Growth rate also correlates imperfectly with killing activity in *E. coli* when grown in a minimal medium with a panel of nutrient sources,^167^ and a recent study has suggested that metabolic state (i.e., ATP availability) is more predictive of antibiotic‐mediated killing than growth rate.^168^ Likely, it is thus not growth/division rate per se that modulates killing by cell wall–active agents, but rather the specific nature and dynamics of PG turnover processes, which are imperfectly correlated with growth rate. Consistent with this idea, several β‐lactams have been identified that do kill nongrowing cells. Imipenem and some experimental β‐lactams can induce lysis in stationary phase cultures of *E. coli* and those that have stopped growing due to nutrient starvation.^169^, ^170^, ^171^ This might be due to the carbapenem's ability to inhibit l,d transpeptidases.^172^ In some bacteria, DAP–DAP crosslinks are upregulated under nongrowth conditions like stationary phase,^45^, ^173^ and at least in *Mycobacterium tuberculosis*, l,d TPs (rather than PBPs) contribute significantly to PG integrity maintenance.^174^ Lysis of nongrowing *E. coli* cells by carbapenems might reflect a similar enhanced reliance on l,d TPs under nongrowth conditions. Importantly, lysis protection by slow growth may also be overcome by simply increasing the concentration of β‐lactams,^146^ which may provide potential lessons for the treatment of infections caused by tolerant bacteria (see discussion below).

The complications surrounding slow growth and antibiotic tolerance are probably best illustrated by research on the SR. The SR is typically induced under conditions of nutrient limitation: amino acid, carbon or fatty acid starvation.^175^ Starvation is sensed by second messenger synthetases like RelA and SpoT. Upon sensing limitation of essential cellular building blocks via interaction with central hubs of metabolism like the ribosome (RelA), these proteins synthesize the second messenger guanosine penta/tetraphosphate ((p)ppGpp, henceforth, “ppGpp” for brevity). Accumulation of ppGpp results in massive transcriptional reprogramming of the cell,^176^ and, most importantly, induces transient growth arrest^177^, ^178^, ^179^ (this is the basis for the well‐established technique to isolate auxotrophic mutants using penicillin enrichment^180^, ^181^). Not surprisingly, the SR has been implicated in tolerance to CWA antibiotics in numerous studies with both Gram‐positive and ‐negative bacteria,^178^, ^182^, ^183^, ^184^, ^185^, ^186^, ^187^, ^188^ and in some cases, clinical mutants with enhanced tolerance due to background SR upregulation have been isolated.^189^ In a particularly intriguing example, the ppGpp synthetases RelP and RelQ of *S. aureus* are induced by antibiotic exposure itself, resulting in high ampicillin and vancomycin tolerance.^135^ The more interesting question surrounding the SR and antibiotic tolerance, however, is whether or not growth arrest is the only effector of SR‐induced tolerance.

A particularly counterintuitive early observation was that in *E. coli*, SR‐induced tolerance could be prevented (and lysis reinstated) by the addition of CHL to amino acid–starved cells.^186^, ^187^, ^190^ CHL addition was proposed to “relax” SR cells to prevent SR induction. Since SR induction by amino acid starvation depends on the interaction of uncharged tRNAs with the ribosome,^191^ inhibiting ribosome function could interrupt the continuous induction of RelA; ppGpp might then be rapidly degraded by the hydrolase SpoT, resulting in the resumption of ppGpp‐inhibited processes. CHL was used at high, growth‐inhibitory concentrations in SR experiments and the observation of CHL‐induced lysis in SR‐induced, β‐lactam–treated cells thus suggested that slow or nongrowth was not the only factor promoting SR‐induced tolerance.

How might SR protect cells from CWA antibiotic–mediated lysis independent of growth arrest? Early work from Edward Ishiguro's group and others suggested that ppGpp accumulation correlated with a decrease in PG synthesis functions^192^ (possibly at a step distal to precursor synthesis^193^, ^194^), and concomitant reduction in PG incorporation. These observations suggested that SR not only induced growth arrest, but also modulated PG synthesis and potentially turnover functions. Consistent with this idea, SR induction resulted in changes in PG architecture.^195^ Further, an increase in both RelA and l,d TPase activity is for an unknown reason required to reprogram *E. coli* cells from majority DAP‐d‐Ala to majority DAP–DAP crosslinks,^196^ also suggesting a PG modulatory function for the SR. Lastly, a potentially direct connection between the SR and cell wall turnover was described in *S. pneumoniae*, where amino acid deprivation changed the cell surface concentration of the autolysin LytA.^185^ As for the mechanistic link between SR and PG turnover, some studies suggested an involvement of fatty acid synthesis in the lysis process, since addition of the fatty acid inhibitor cerulenin could once again protect amino acid–starved, CHL‐treated cells from lysis.^194^, ^197^ As a caveat, cerulenin might have simply reverted these cells to a high ppGpp state, as fatty acid starvation was subsequently shown to induce SR.^183^, ^198^, ^199^ However, a connection between phospholipid synthesis and SR‐mediated ampicillin tolerance was also suggested by another experiment: Overexpression of the glycerol‐3‐phosphate acetyltransferase PlsB (the first dedicated step toward membrane phospholipid synthesis^200^ and a target of ppGpp^201^) reversed SR‐induced tolerance, suggesting that ppGpp‐mediated shutdown of PlsB (overcome by overexpressing it) might be a key step in tolerance development.^188^ In apparent contradiction to all that has been presented above, a recent study found no reduction in PG incorporation (and turnover after antibiotic treatment) in RelA overexpression strains when correcting for reduced growth rate,^202^ and the degree to which SR exerts a direct effect on PG turnover thus remains unclear. Taking all available evidence together, the exact mechanistic connection between SR induction and CWA antibiotic tolerance independent of a growth‐inhibitory effect thus remains poorly understood and might vary with bacterial species.

### Genetic underpinnings of tolerance: loss of autolysin activity

A standard procedure in bacterial genetics is to generate mutants either defective or enhanced in a phenotype to interrogate its mechanistic underpinnings. This approach has been applied to tolerance research as well, both in the laboratory and by exploiting the power of natural selection (e.g., through the study of clinically selected mutations).^203^, ^204^ However, the analysis of mutants for tolerance pathways is not always straightforward. Highly tolerant mutants often include those with defects in important metabolic processes that probably confer CWA antibiotic tolerance solely as a secondary consequence of the slow growth rate associated with these mutations. For example, the small GTPase RsgA of *S. aureus* is required for ribosome assembly. An *rsgA* mutant exhibits reduced growth rate and is thus more tolerant to penicillin.^205^ Similarly, mutations in amino acid homeostasis (isoleucine tRNA synthetase) exhibit apparent vancomycin tolerance via slow growth.^206^ These types of mutants are thus less informative to elucidate mechanisms of tolerance, though they do often emphasize the significance of slow growth for tolerance development.

Early research using hypertolerant (both laboratory and clinically derived) Gram‐positive bacteria (*S. pneumoniae, S. aureus, B. subtilis*, and *Lactobacillus* species) led to the conclusion that these variants were defective in major autolysin activity,^88^, ^115^, ^120^, ^207^, ^208^ either due to direct mutational inactivation of cell wall hydrolases,^204^, ^209^, ^210^ by altering PG structure (potentially changing the substrate of autolysins)^195^, ^211^ or by disruption of regulatory pathways upstream of autolysin activity.^88^ For example, some highly tolerant mutants identified in Gram‐positive bacteria produced higher levels of TAs,^204^, ^212^ which can inhibit autolysin activity,^23^ by sequestration^118^ and/or protective coating of otherwise vulnerable PG species.^122^

In many cases, the regulatory pathways upstream of autolysin activity are still incompletely understood. Mutants in the *lrgAB* versus *cidAB* loci of *S. aureus*, for example, had opposing effects on tolerance to penicillin. In the absence of *lrgAB*, autolysin activity was high, and tolerance consequently low.^213^ In the absence of *cidAB*, autolysin activity was low, and tolerance was 100‐fold higher than the parental strain.^214^ LrgA and CidA exhibit similarities to antiholin/holin proteins of bacteriophages,^215^ and it was proposed that CidA (holin homolog) is somehow required for autolysin export, which is opposed by the antiholin homolog LrgA. The physiological function of these systems has been proposed to be DNA release during biofilm formation,^216^ but how they contribute to autolysin regulation is still unknown. What is conspicuously missing from the list of examples above are Gram‐negative bacteria. Indeed, it seems to be challenging to generate mutants in autolysins or their regulators that confer high tolerance, but this might simply be a consequence of using *E. coli* for most experiments, which is among the least tolerant Enterobacterales for unknown reasons.^83^ The relative paucity of autolysin‐deficient, highly tolerant mutants might be due to their thinner PG layer, which would not allow for the same degradation buffering capacity compared with the thicker Gram‐positive cell wall, or due to some differences in autolysin activity or regulation (e.g., a higher degree of functional redundancy in key autolysins in Gram‐negatives).

### Nongenetic mechanisms of tolerance: stress response systems and damage repair

As described above, cell wall–acting agents tend to cause massive cell damage and thus induce complex responses. In principle, bacteria can become tolerant by suppressing those responses that are harmful to the cell, and/or by recognizing and repairing damage induced by the antibiotic (inducible responses promoting tolerance are summarized in Table 1). Bacterial responses to the environment are typically mediated by dedicated two‐component systems, alternative sigma factors and other stress‐sensing regulators, which sense specific signals (e.g., cellular damage) and induce damage repair regulons.^217^ Many stress‐sensing systems are induced by exposure to CWA antibiotics, and the diversity of transcriptional responses to these agents reflects the diversity of cell damage induced by CWA antibiotics. Not surprisingly, stress responses often contribute to survival (and thus tolerance) in the presence of CWA antibiotics. The cell envelope stress response alternative, sigma factor RpoE, for example, is activated by CWA antibiotics and required for β‐lactam tolerance in *Burkholderia pseudomallei* and *V. cholerae*.^148^, ^165^ RpoE controls general cell envelope health functions, such as periplasmic proteases and chaperones,^218^ and likely contributes to cell envelope strength when the cell wall is weakened. The stationary phase alternative sigma factor RpoS contributes to imipenem tolerance in *P. aeruginosa*,^219^ perhaps due to its involvement in maintaining the OM permeability barrier,^220^ or through its positive control over the superoxide dismutase SodB.^221^

**Table 1:** Inducible mechanisms of CWA tolerance

Species	Signal	Tolerance genes	Proposed tolerance mechanism	Citations
*Streptococcus pneumoniae*	Unknown	Unknown	Production of teichoic acids and downregulation of autolysin activity	204, 212
	Vancomycin	*ciaRH*	Downregulation of autolysin activity	232
	Vancomycin	*ptvR*	Unknown	235
*Streptococcus pyogenes*	Unknown	*Stk*	Unknown	230
*Staphylococcus aureus*	Unknown	Unknown	Production of teichoic acids and downregulation of autolysin activity	204, 212
	Unknown	*lrgAB/cidAB*	Downregulation of autolysin activity	213, 214
	β‐lactam exposure	*relP/relQ*	Slow growth	135
	Unknown	*sodA*	Detoxification of ROS	
*Enterococcus faecalis*	Unknown	*sodA*	Detoxification of ROS	237
*Bacillus subtilis*	Cell wall fragments	*walRK*	Inhibition of autolysin activity through IseA	227
*Escherichia coli*	pH	Unknown	Unknown	138
	d‐amino acids	Unknown	Unknown	140
	Cell envelope/wall damage	*Rcs*	Unknown	225
*Vibrio cholerae*	CWA exposure and autolysin overexpression	*vxrAB*	Upregulation of cell wall synthesis and remediation of toxic iron influx	166
	Penicillin exposure/misfolded OMPs	*rpoE*	Cell envelope maintenance	165
	Penicillin exposure	*sodB*	Detoxification of ROS	128, 165
*Pseudomonas aeruginosa*	Starvation/biofilm growth/stationary phase	*sodB*	Detoxification of ROS	221, 238
	Entry into stationary phase	*rpoS*	Unknown	219
*Burkholderia pseudomallei*	Unknown, likely CWA exposure	*rpoE/degS*	Unknown	148
Multiple species	Nutrient starvation	*relA/spoT*	Slow growth/reduced PG turnover	180, 181

The most well‐studied pathogen in the context of Gram‐negative extreme β‐lactam tolerance is *V. cholerae*. Here, the VxrAB two‐component system is the major contributor to tolerance. VxrAB is induced by various forms of cell wall damage (CWA antibiotics and overexpression of endogenous PG EPs).^166^ A ∆*vxrAB* mutant exhibits ∼10,000‐fold decreased tolerance against CWA antibiotics compared with the wild‐type strain; importantly, this is not the consequence of enhanced lysis, but rather reflects reduced recovery of cell wall–deficient spheroplasts that are formed in response to CWA antibiotics in this organism.^17^ VxrAB positively controls genes encoding cell wall synthesis enzymes (including the entire intracellular precursor pathway), type VI secretion, and biofilm formation, and negatively controls motility and iron acquisition genes.^128^, ^166^, ^222^, ^223^ Both positive control over PG synthesis and negative control over iron acquisition contribute to CWA antibiotic tolerance.^128^ While PG synthesis induction is an intuitive way for a cell to recover from cell wall loss, control of iron acquisition was at first puzzling. Intriguingly, *V. cholerae* accumulates high intracellular iron levels upon β‐lactam exposure and this is exacerbated in the ∆*vxrAB* mutant.^128^ Further, β‐lactam exposure resulted in induction of the Fur iron starvation response, likely due to direct oxidation of its Fe^2+^ corepressor by β‐lactam–induced H_2_O_2_ production.^128^ It thus appears that VxrAB's role in tolerance is at least partially to tone down an out‐of‐control iron starvation response that would otherwise overload β‐lactam–stressed cells with iron, causing downstream toxic events like the generation of ROS. Consistent with this model, deleting iron transporters partially restored high tolerance levels to a ∆*vxrAB* mutant.^128^ The VxrAB system thus aptly illustrates the complex physiological consequences of β‐lactam exposure and tolerance pathways, where cells not only have to overcome cell wall loss, but also remediate toxic iron influx and potentially downstream accumulation of toxic ROS.

While the VxrAB system is only conserved among *Vibrionaceae*, other pathogens have potentially functionally analogous cell envelope stress response systems with roles in CWA antibiotic tolerance. The enterobacterial Rcs phosphorelay system negatively controls motility^224^ and is required for recovery from a cell wall–deficient state in *E. coli*,^225^ which is reminiscent of VxrAB phenotypes. In *B. subtilis*, the WalRK two‐component system plays a central role in cell wall homeostasis under normal growth, but also under CWA antibiotic–induced stress conditions.^226^ WalRK controls IseA/YoeB, a post‐translational inhibitor of EP activity, which effectively downregulates lytic activity upon exposure to CWA antibiotics.^227^ Interestingly, IseA is induced through WalRK specifically in response to the accumulation of PG degradation products resulting from d,l EP activity,^228^ which are likely to accumulate in CWA‐stressed cells. The Serine/Threonine protein kinase Stk that putatively feeds into the WalRK signaling cascade^229^ has been implicated in penicillin tolerance in *S. pyogenes*,^230^ which might further suggest a conserved role for WalRK in CWA antibiotic tolerance. In *S. pneumoniae*, the CiaRH two‐component system promotes tolerance to bacitracin, d‐cycloserine, and vancomycin.^231^, ^232^ This system is induced by CWA antibiotics (vancomycin)^130^ and controls several small RNAs^233^ with unclear targets. However, one of the consequences of activation is reduced autolysis through a process that requires capsular polysaccharide biosynthesis.^232^, ^234^ Lastly, and similarly poorly understood, *S. pneumoniae* also encodes the PtvR transcriptional repressor, which controls a regulon that slightly (∼5‐ to 10‐fold) enhances vancomycin tolerance upon sensing the antibiotic.^235^

In addition to obvious cell envelope stress functions, some less intuitive responses are also induced by CWA antibiotics, and these may similarly contribute to tolerance. Reactive oxygen species accumulate upon exposure to CWA antibiotics, purportedly due to an imbalance of respiratory processes^125^, ^129^, ^236^ that results from large‐scale perturbations of cell wall metabolism. ROS can potentially contribute to CWA antibiotic–induced lethality, and detoxification of ROS promotes CWA antibiotic tolerance in some bacteria; for example, superoxide dismutases (SODs) are effectors of β‐lactam tolerance in *Enterococcus faecalis, V. cholerae*, and *Pseudomonas aeruginosa*.^165^, ^221^, ^237^ In *P. aeruginosa*, a convergence of three stress response functions (SR, ROS detoxification, and an alternative sigma factor) promotes tolerance during growth in biofilms: the SR (see above) was implicated in tolerance to carbapenems and other antibiotics via its positive control over the Mn‐dependent SodB.^238^ A mutant incapable of producing ppGpp survived ∼10,000‐fold less than the wild‐type in the presence of high meropenem concentrations and an *sodB* mutant recapitulated this phenotype.^221^ Additionally, *sodB* was coregulated by the stationary phase alternative sigma factor RpoS. Overall, these observations suggest that an ability to detoxify ROS that accumulate after antibiotic exposure may be an important component of tolerance development. This is contrasted by observations in divergent bacteria that suggest ROS can actually promote the formation of persister cells, that is, a hypertolerant subpopulation.^239^, ^240^ It is, therefore, possible that ROS have contrary effects on heterogeneous pathogen populations and either promote or reduce tolerance/persistence depending on the specific physiological state or growth environment bacteria are in upon antibiotic exposure. Consistent with a growth modality component, the requirement for SODs appears to be most prominent during stationary phase or biofilm growth in both *V. cholerae* and *P. aeruginosa*,^128^, ^221^ rather than during exponential growth.

In both *E. coli* and *S. aureus*, exposure to β‐lactams results in induction of the SOS DNA damage response.^132^, ^133^ In *E. coli*, this is mediated specifically by inhibition of PBP3 in a sensing process requiring the two‐component system DpiAB.^133^ SOS induction results in inhibition of cell division through the FtsZ antagonist SulA. Since cell division is typically the location where β‐lactam–mediated lysis starts, at least in *E. coli*,^145^ preventing septum formation might delay lysis and thus promote temporary tolerance.

For unknown reasons, the heat shock response (via the alternative sigma factor RpoH) is induced by CWA antibiotics in phylogenetically diverse bacteria,^130^, ^131^, ^241^, ^242^, ^243^ suggesting a conserved pathway for induction. The signal for heat shock might be protein misfolding due to oxidation, which has been reported to occur in after exposure to β‐lactam antibiotics.^128^, ^129^ The exact degree to which heat shock contributes to tolerance is poorly understood; however, artificially upregulating RpoH prevented β‐lactam–induced lysis in *E. coli*,^244^ suggesting a potential role in surviving β‐lactam exposure. Further, in *S. pneumoniae*, the heat shock–induced protease ClpL enhances tolerance to oxacillin, likely by stabilizing the expression of bPBP2x,^131^ thus, promoting cell wall integrity maintenance.

## Part III. Tolerance and antibiotic therapy

### A special case of CWA antibiotic tolerance: L‐forms and spheroplasts

**Figure 3:**
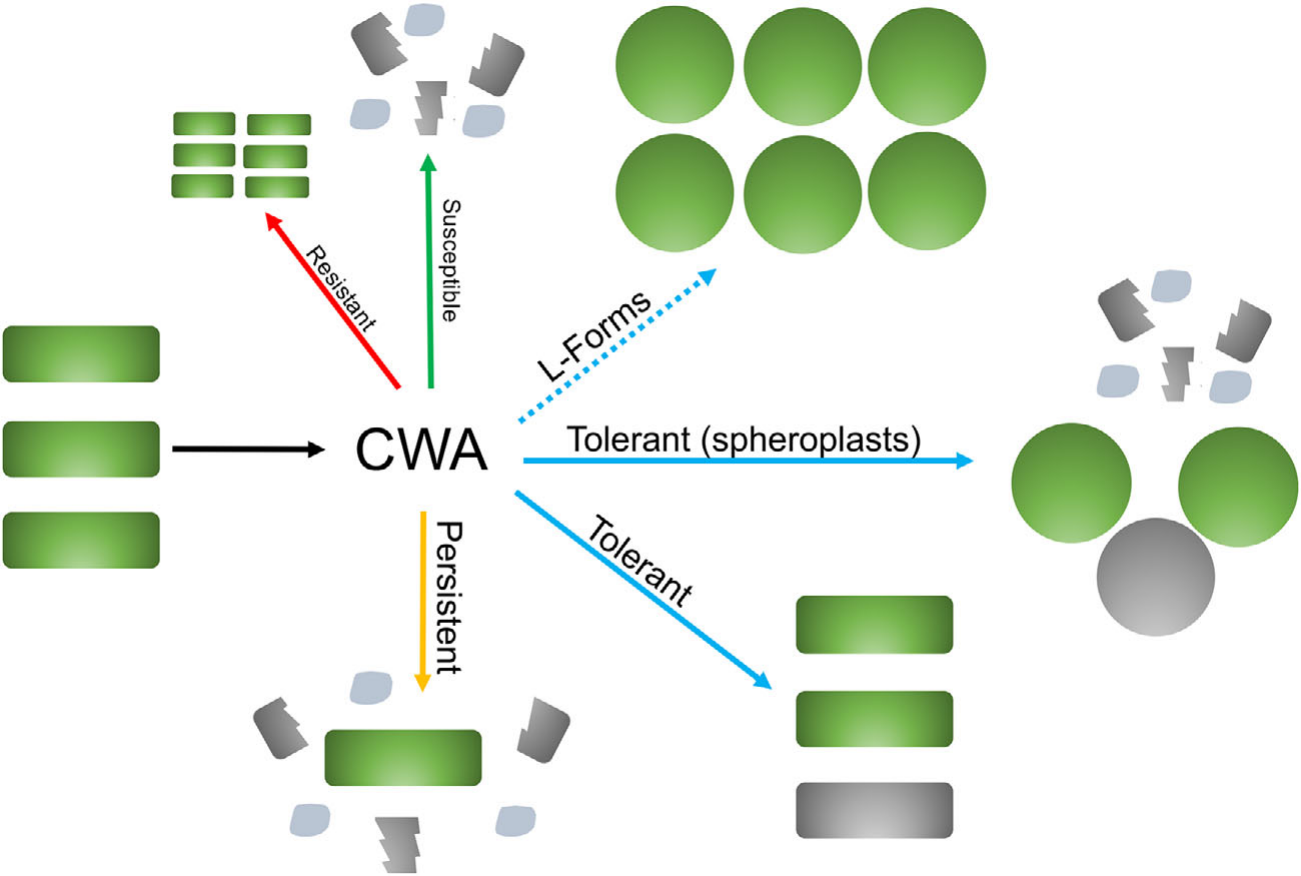
Variations of the morphological consequences of CWA antibiotic exposure. CWA antibiotics induce cell wall degradation in most growing bacteria. Resistant isolates can grow in the presence of these antibiotics. Susceptible, nontolerant populations lyse and die, while rare persisters may remain intact. In the absence of resistance mechanisms and in addition to persisters, CWA antibiotic–treated bacteria can present as L‐forms (Gram‐positive and ‐negative bacteria) that are able to divide in the absence of a cell wall, nondividing and cell wall–deficient spheroplasts (Gram‐negative bacteria), or tolerant cells of unknown morphology (here depicted as rods). Living cells are shaded in green; dead cells and debris are in gray.

While shutting down growth is an effective means to resist antibiotic killing, some bacterial species and environmental conditions favor a different fate. Many bacteria are capable of fully enduring the destructive effects of CWA antibiotic exposure by converting to viable, cell wall–deficient forms (Fig. 3). Cell wall–deficient L‐forms (extensively and excellently reviewed elsewhere, see Refs. 84, 86, and 245) circumvent PG essentiality by proliferating via FtsZ‐independent stochastic membrane blebbing.^246^, ^247^ In addition, many Gram‐negative bacteria are capable of converting into a nondividing, cell wall–deficient spheroplast state upon β‐lactam exposure. Importantly, spheroplasts are distinct from the spherical cells that emerge upon inhibition of bPBP2. While the latter superficially resemble spheroplasts, they actually elaborate a full cell wall,^83^ while true spheroplasts are devoid of detectable PG material,^17^, ^83^ and thus presumably rely on the strength of their outer membrane to maintain structural integrity. The ability to do so appears to be widespread among clinically significant Gram‐negative pathogens (by current count *Acinetobacter baumannii, Enterobacter* species, *Haemophilus influenzae, Klebsiella* species, *P. aeruginosa*, *Serratia marcescens*, and *V. cholerae* ^17^, ^83^, ^85^, ^87^, ^248^) and is observed under *in vivo*‐like conditions (e.g., rabbit cecal fluid and human serum).^17^, ^83^ In *V. cholerae* and some Enterobacterales isolates, spheroplast formation results in an extreme form of tolerance, essentially converting β‐lactams to bacteriostatic antibiotics against these bacteria.^17^, ^83^, ^165^ Spheroplasts readily and quickly revert to rod‐shaped bacteria upon withdrawal of the antibiotic,^83^, ^87^ which would potentially enable them to repopulate an infection upon depletion of the offending antibiotic. Importantly, L‐forms and spheroplast‐like forms have been isolated from patients treated with CWA antibiotics,^249^, ^250^ and this form of tolerance might thus be an under‐recognized reason for antibiotic treatment failure.^250^ The exact mechanisms of spheroplast formation and maintenance are poorly understood, but physiological and genetic factors promoting this type of tolerance have been identified.^87^, ^128^, ^165^ In *V. cholerae*, survival of, and recovery from, the spheroplast state requires induction of PG synthesis functions, remediation of toxic iron influx, likely detoxification of ROS, and induction of cell envelope stress responses, as detailed above.^128^ An intriguing unresolved problem is how β‐lactam–tolerant spheroplasts survive futile cycling^99^ induced by β‐lactams and what makes their OM particularly resilient compared with nontolerant (lysis‐prone) Gram‐negatives.

### Clinical relevance of CWA antibiotic tolerance

Intuitively, being able to survive exposure to normally lethal antibiotics might cause treatment failure if the offending pathogen is able to regrow after discontinuation of antibiotic therapy. However, just inhibiting pathogen growth is typically sufficient to give the immune system time to clear an infection. Whether for most infections bactericidal antibiotics perform superiorly to bacteriostatic ones is thus a matter of contention,^251^, ^252^ though in at least some cases they clearly do (e.g., in staphylococcal septicemia^253^). As far as direct treatment outcomes are concerned, tolerance might thus contribute adversely only under special circumstances, for example, in environments that are not easily penetrable for immune functions. Several peculiarities of the tolerance phenomenon compared with resistance (i.e., changes in MIC) have precluded studies rigorously testing clinical hypotheses surrounding tolerance. For example, tolerance can be more condition‐specific than resistance. MICs obtained in routine media used for antibiotic susceptibility testing, such as cation‐adjusted MHB, correlate with treatment outcome *in vivo*,^254^ suggesting that a concentration of antibiotic that inhibits growth of a pathogen in the laboratory is likely to do the same in the human body. Tolerance, on the other hand, varies dramatically with growth in very specific niches, as we have outlined above for *Salmonella* growing in macrophages and *S. aureus* isolated from model tissue cage infections. Removing a bacterium from this specific *in vivo* niche and allowing it to grow under optimal conditions in the laboratory could cause it to revert to a nontolerant state almost immediately. As discussed above, well‐established quantitative tolerance tests that can be incorporated into a clinical microbiology workflow are also lacking, preventing routine testing for tolerance in the healthcare setting. Existing studies that address the clinical impact of tolerance, therefore, suffer from the fact that quantitative definitions of tolerance are often arbitrary and cannot be used to clearly establish causation. Lastly, cryptic resistance mechanisms (e.g., resistance of a subpopulation that is not easily detectable by standard MIC assays) can create the appearance of antibiotic therapy failure in the absence of overt resistance. Treatment failure might in such a situation be interpreted as relying on something other than resistance (i.e., tolerance), while the real culprit might be heteroresistance,^255^ like heterogeneously vancomycin intermediate *S. aureus* (hVISA).^256^

Despite the above‐mentioned complications, numerous attempts have been made to quantitatively determine the extent to which tolerance affects outcome of antibiotic therapy and several animal model infections have supported the importance of tolerance. For example, imipenem treatment failed to clear an experimental *Enterobacter cloacae* thigh infection in a mouse model, despite high susceptibility of *E. cloacae* to the antibiotic.^257^ In a guinea pig model of foreign body infections by *S. epidermidis*, cure rates after vancomycin/teicoplanin therapy correlated more strongly with MBC than with MIC (both determined in MHB),^258^ suggesting that intrinsic (rather than condition‐specific) tolerance can indeed influence infection outcome. Similar results were observed in a rat model of foreign body infections.^157^ In an endocarditis rabbit model, higher tolerance in streptococci correlated with adverse outcome at low, but not high penicillin concentrations.^259^ Lastly, thigh infections in a mouse model using *S. aureus* were cleared at a lower rate when caused by highly tolerant variants compared with wild‐type strains exhibiting lower tolerance.^260^

Clinical evidence for a role of tolerance in antibiotic treatment outcomes remains scarce, although suggestive anecdotes and case reports abound.^253^, ^261^, ^262^, ^263^ Some established infections of *S. aureus* are known for their high rates of treatment failure despite their *in vitro* susceptibility (determined by MIC and MBC), and often require surgical intervention.^263^ Peterson *et al*. reported on three patients with *S. aureus* septicemia or endocarditis, where initial oxacillin, nafcillin, or vancomycin monotherapy failed to yield a favorable treatment outcome as defined by cessation of clinical symptoms and/or negative blood culture.^16^ The MICs and MBCs as determined in standard laboratory media were well below the blood serum levels of the antibiotics (which were measured in the same study), suggesting that clearance failure may be due to tolerance. A similar observation was made in another case study of *S. aureus* endocarditis, where vancomycin therapy failed to treat an infection caused by fully susceptible bacteria;^264^ however, note the discussion of hVISA strains above. Pediatric pharyngitis caused by *S. pyogenes* was found to exhibit a high degree of treatment failure after penicillin therapy, again despite apparent susceptibility of the involved pathogen.^265^ In another example, a prospective study conducted by Elaine Tuomanen's group found that infection with CWA antibiotic–tolerant *S. pneumoniae* isolates correlated with adverse outcomes in pediatric meningitis patients, where survival after treatment with CWA antibiotics was 49% for those infected with tolerant streptococci and 86% in those infected with nontolerant isolates.^266^ Tolerance was defined here via the magnitude of increased survival in the presence of vancomycin compared with the nontolerant reference strain R6.

In further support of the relevance of tolerance in the healthcare setting, CWA antibiotic–tolerant mutants have been isolated from patients after antibiotic therapy.^267^, ^268^, ^269^ The vancomycin‐tolerant *S. pneumoniae* Tupelo isolate was obtained from a patient with meningitis in which vancomycin/cephalosporin treatment had failed despite full vancomycin susceptibility of the isolate.^270^ Interestingly, this isolate was later found to contain a point mutation in the histidine kinase CiaH, which was hypothesized to activate the tolerance‐promoting *ciaRH* regulon (see above for details).^234^ Other vancomycin‐tolerant *S. pneumoniae* isolates have been tied to specific capsular serotypes,^271^ potentially supporting the poorly understood connection between capsule biosynthesis and tolerance, possibly via CiaRH.^234^ As is apparent from these examples, most clinical studies on tolerance have focused on the classical Gram‐positive model organisms of tolerance research, namely, members of the genera *Staphylococcus* and *Streptococcus*. Much less is known about the clinical significance of tolerance for Gram‐negatives, though case studies suggest instances of similar unexplained treatment failure despite the organism's *in vitro* susceptibility to antibiotics.^249^, ^261^, ^272^

Conversely, some studies have failed to show a clear correlation between tolerance and clinical outcomes; however, these studies are difficult to interpret due to their use of several antibiotics and the aforementioned problems with tolerance definitions and measurements. Mortality of a pediatric population with *S. aureus* bacteremia correlated more strongly with comorbidities than with tolerance (defined as MBC/MIC >10),^212^ but these infections were treated with combination therapy. Another study found no strong correlation between tolerance (defined as high MBC) and oxacillin/glycopeptide treatment outcomes in patients suffering from endocarditis caused by *S. aureus*.^273^

Overall, the connection between tolerance and treatment outcome is thus data deficient and requires—and deserves—further investigation. In addition, clinical studies have focused mainly on a direct influence of tolerance on clinical outcomes and neglected what is most likely a nefarious side effect of tolerance: its role as a stepping stone toward development of frank resistance. Several studies have shown that tolerant cells are a reservoir for the emergence of resistant variants.^274^, ^275^, ^276^, ^277^ This is at least in part due to the simple numerical fact that swiftly eradicating a pathogen population provides a lower chance of emergence of rare resistant variants. Furthermore, tolerant, damaged cells can actually exhibit enhanced mutation frequencies,^278^, ^279^ and this might be particularly true for CWA antibiotic–induced spheroplasts, which are metabolically active and accumulate oxidative damage. Thus, it is imperative that efforts be directed toward devising novel therapies aimed at eradicating tolerant pathogens, either through a combination therapy approach or via the development of novel antibiotics. The latter might be achieved through a recently developed, innovative antibiotic screening platform that relies on indicator‐enabled scoring of lethality in bacteria grown to stationary phase on filter disks.^280^ It seems that in some cases, CWA agent tolerance can be overcome by simply increasing the concentration of antibiotic, as even slow‐growing bacteria can be killed at higher concentrations of CWA agents.^146^ Optimization of antibiotic treatment length and dose during antibiotic therapy thus likewise hold great potential to improve the efficacy of existing antibiotics against tolerant cells.^281^, ^282^

## Competing interests

The author declares no competing interests.
